# Impending extrusion of Ex-PRESS shunt treated by shunt-position adjustment: a case report

**DOI:** 10.1186/s12886-017-0665-2

**Published:** 2018-01-08

**Authors:** Yong Ju Song, Sumi Kim, Gil Joong Yoon

**Affiliations:** 1 Department of Ophthalmology, Chosun University College of Medicine, Gwangju, South Korea; 2 Department of Dentistry, Hallym University Gangnam Sacred Heart Hospital, Seoul, South Korea; 3 Happy Eye21 Hospital, 950, Mujin-daero, Seo-gu, Gwangju, 61932 Republic of Korea

**Keywords:** Ex-PRESS shunt, Shunt extrusion, Shunt malposition, Shunt-position adjustment

## Abstract

**Background:**

To report a case of impending extrusion of Ex-PRESS shunt treated by shunt-position adjustment.

**Case presentation:**

A 56-year-old Asian woman presented with impending extrusion and malposition of Ex-PRESS shunt in her left eye. The bleb of the left eye was shallow and diffuse. In the past, the patient was treated by Ex-PRESS shunt implantation under the scleral flap in both eyes. There had been no Ex-PRESS shunt-related complication in her right eye, and she reported no history of left-eye trauma. Based on these findings, we hypothesized that the source of the left-eye problem was a loosely fixed Ex-PRESS shunt spur. It was thought, furthermore, that this inadequate scleral resistance during the Ex-PRESS shunt implantation was due to the low scleral rigidity resulting from high myopia and insufficient maintenance of the anterior chamber. We proceeded to make an incision in the area adjacent to the Ex-PRESS shunt using a super sharp blade. The shunt was then pushed into the anterior chamber with forceps, and the spur was fixed firmly. Pushing the shunt to the anterior chamber was found to have been sufficient to fix it firmly. In fact, when the sclera was palpated with a sponge, aqueous outflow was observed with no shunt displacement. Postoperative intraocular pressure (IOP) was managed well, and the bleb had formed with diffuse, prominent shapes. The Ex-PRESS shunt was well sustained with good positioning.

**Conclusions:**

When an Ex-PRESS shunt operation is performed on a patient who shows a tendency for low scleral rigidity, shunt implantation should be accomplished carefully and with force adequate for firm spur fixation.

## Background

The Ex-PRESS shunt (Alcon Laboratories Inc., Fort Worth, TX) is a stainless steel non-valve tube. It has a spur designed for fixation in the trabecular meshwork without suturing. Originally implanted under the conjunctiva to connect the anterior chamber to the subconjunctival space [1], it is now positioned under the scleral flap, due to complications such as hypotony, conjunctival erosion, shunt extrusion, and exposure resulting from the earlier methodology [2–4].

In order to regulate intraocular pressure (IOP) adequately and prevent complications including shunt extrusion or exposure, the Ex-PRESS shunt must be properly positioned and securely fixed. There have been reports of Ex-PRESS shunt removal necessitated by shunt extrusion or exposure resulting from improper fixation or trauma, or simply by spontaneous extrusion or exposure [4–7]. We herein report a case of impending improper-fixation-caused extrusion of Ex-PRESS shunt treated by shunt-position adjustment without shunt removal.

## Case presentation

**Fig. 1:**
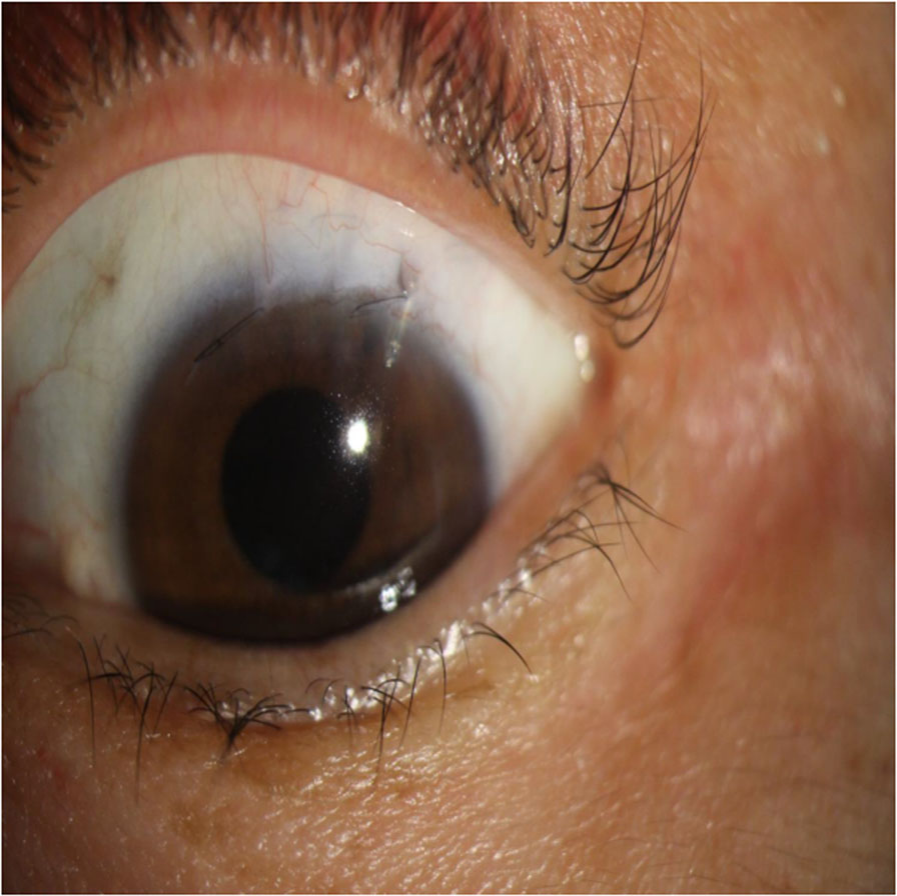
Ex-PRESS shunt showing impending extrusion. The internal opening was tilted to the corneal endothelium obliquely in the anterior chamber, and the external plate was prominent in the subconjunctival space

A 56-year-old Asian woman visited our clinic. Her best-corrected visual acuity (BCVA) was 20/20, and the IOP in the problem eye (the left) was 13 mmHg. Slit-lamp examination showed impending Ex-PRESS shunt extrusion or exposure. The internal opening was tilted to the corneal endothelium obliquely in the anterior chamber, and the external plate was prominent in the subconjunctival space (Fig. 1). The color of the bleb was pale, and its shape was diffuse and shallow, though there was no microcyst.

The patient had been followed up on for senile cataract and open-angle glaucoma, with maximal medical therapy. Seven (7) months previously, IOP in both eyes was uncontrolled, ranging from 25 to 28 mmHg. The spherical error was −9.5 diopters(D) and axial length (AXL) was 26.98 mm in the right eye. The spherical error was −5.0D and AXL was 27.06 mm in the left eye. BCVA was 20/200 in the right eye and 20/25 in the left eye, due to nuclear cataracts. This had necessitated combined surgery (cataract + Ex-PRESS shunt implantation) in our hospital. A 3 × 3 mm scleral flap of half thickness was made at superotemporal area. Then, phacoemulsification and lens implantation was performed through a 2.2 mm corneal incision site located in the temporal area. The tract was made with a 25-gauge needle in one-third of the medial area of the blue-gray transition zone, under a preformed scleral flap. The Ex-PRESS shunt (P200; Alcon Laboratories Inc., Fort Worth, TX) was then pushed into the anterior chamber. After surgery, right-eye visual acuity, IOP, bleb shape and Ex-PRESS shunt positioning were all satisfactory. In the left eye, however, the spur of the Ex-PRESS shunt was not firmly fixed in the trabecular meshwork, and the shunt had been implanted obliquely. These left-eye problems were thought to have been caused by low scleral rigidity resulting from high myopia and insufficient maintenance of the anterior chamber, which presumably had rendered scleral resistance inadequate during Ex-PRESS shunt implantation. We decided to observe carefully without surgical intervention, because the external plate of the shunt was covered well by scleral flap and there was no extrusion sign.

**Fig. 2:**
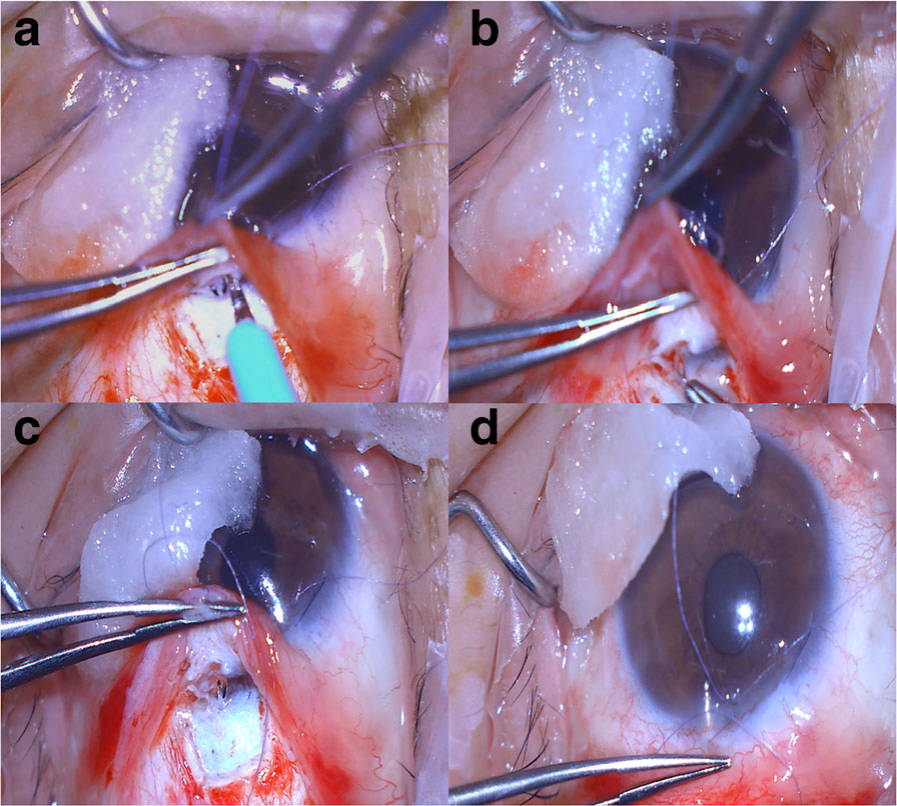
Process of Ex-PRESS shunt-position adjustment. **a** Incision into anterior chamber parallel to iris plane, in both lateral directions, from external-plate-inserted site. **b** Ex-PRESS shunt grasped and pushed into anterior chamber with forceps. **c** External plate of shunt tight on sclera. **d** Internal opening of shunt parallel to iris

**Fig. 3:**
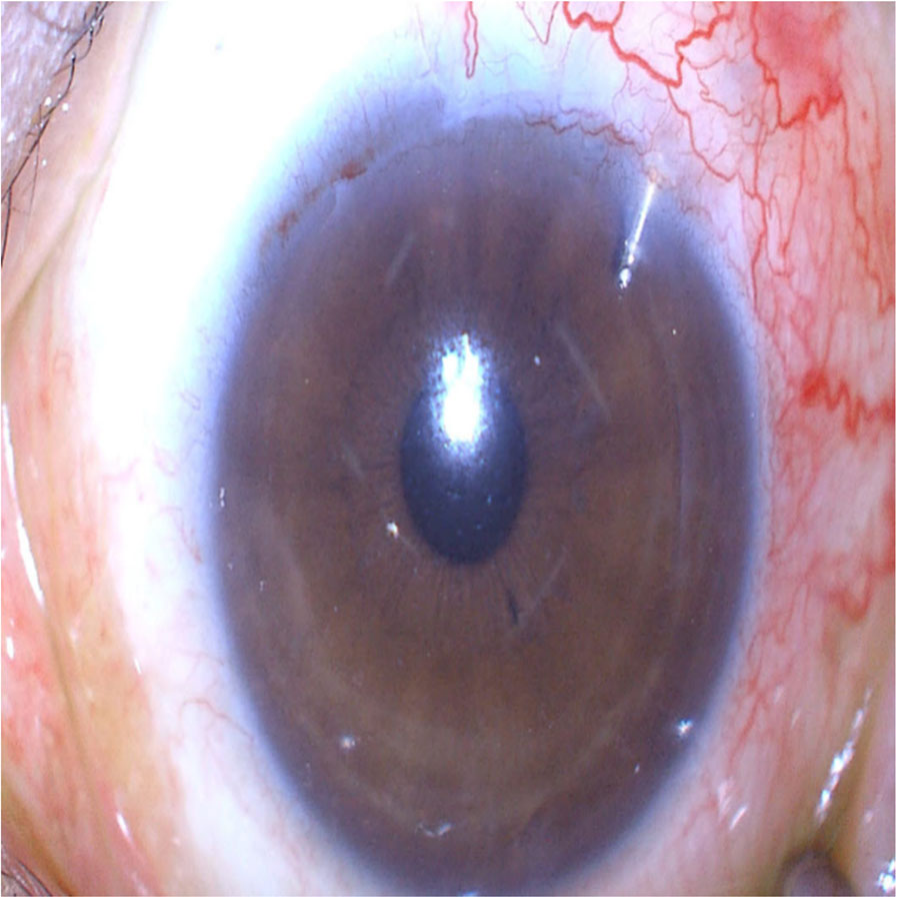
Postoperatively, Ex-PRESS shunt was sustained with good positioning

Seven (7) months postoperatively, we observed an impending extrusion sign that the external plate was prominent in the subconjunctival space, though it was covered by the scleral flap. Because Ex-PRESS shunt exposure can necessitate shunt removal or additional operation for IOP control, surgery was performed prior to any exposure. A limbus-based conjunctival incision was made followed by careful dissection of the adhesions between the subconjunctival and Tenon tissue. A subsequent incision was made along the previous scleral-flap wound with a super sharp blade, and then the scleral flap was opened carefully until the Ex-PRESS shunt was visible. After dissection of adhesions between the external plate of the EX-PRESS shunt and the surrounding tissue was performed, the shunt was grasped with forceps. However, as the shunt was fixed stronger than expected and, thus, was not easily removable by forceps, we decided against removal and opted for adjustment of its position instead. To make sufficient space for easy adjustment, an incision with the super sharp blade was made from the external-plate-inserted site, in both lateral directions, into the anterior chamber parallel to the iris plane. Then, the shunt was inserted perpendicularly to the limbus, parallel to the iris plane, and well pushed in anterior chamber by forceps for firm fixation of the spur. The internal opening was parallel to the iris, and the external plate was tight on the sclera (Fig. 2). When sclera was palpated with a sponge, aqueous outflow was observed without shunt displacement. After checking repeatedly, the scleral flap was closed with 4 simple sutures. Mitomycin C (MMC) 0.04% was applied for 4 min, followed by irrigation. The MMC was applied as far as possible from the distal margin of the scleral flap, so as to minimize the possibility of MMC penetration into the anterior chamber. Finally, the conjunctival incision was sutured. Postoperatively, IOP was maintained between 10 and 12 mmHg and the bleb was limited to the diffuse, prominent form. The Ex-PRESS shunt was sustained with good positioning (Fig. 3).

## Discussion

Glaucoma is a leading cause of irreversible blindness worldwide [8]. If IOP cannot be controlled with medication, surgery is necessary. The standard procedure has been trabeculectomy. For better trabeculectomy results, the Ex-PRESS shunt (Optonol Ltd., Neve Ilan, Israel) was introduced by Belkin and Glovinsky in 1998 [9]. It is a stainless steel, non-valve tube composed of a body, spur and external plate. Currently, a device of 2.64 mm length and 50 or 200 um internal diameter (P50 or P200; Alcon Laboratories Inc., Fort Worth, TX) is commonly used [10]. The Ex-PRESS shunt connects the anterior chamber and subconjunctival space. Unlike conventional trabeculectomy, Ex-PRESS shunt surgery can be formulated. In trabeculectomy, the sizes of the fistula and corresponding iridectomy vary by operator’s preference. However, the size of lumen of the Ex-PRESS shunt is standardized and as such, it is possible to estimate the filtration amount. And Ex-PRESS shunt surgery does not require sclerectomy or iridectomy. Compared with trabeculectomy in theologically, it can decrease inflammation and hyphema [11]. Moreover, a meta-analysis reported that Ex-PRESS shunt surgery showed similar efficacy to conventional trabeculectomy in terms of IOP lowering, vision recovery and operative success rate [12]. Given these advantages, Ex-PRESS shunt surgery has been utilized widely in the treatment of glaucoma.

In the early days of its application, the Ex-PRESS shunt was implanted under the conjunctiva; however, complications such as hypotony, conjunctival erosion, shunt extrusion or exposure were encountered. To prevent such complications, Dahan and Carmichael performed implantation under the scleral flap [13]. In the decade since this innovation, complications have been rare. Among Stein JD et al.’s 8 documented cases (8 eyes) of Ex-PRESS shunt exposure, only 2 had been implanted under the scleral flap (the 6 others, under the conjunctiva) [6]. In each case, the shunt was removed and a scleral patch graft was performed to close the residual 400 um diameter wound. Kourin AS et al. reported one case of Ex-PRESS shunt scleral-flap implantation necessitating shunt removal [7]. Performing a 2-mm incision adjacent to the shunt and penetrating the anterior chamber parallel to iris, they grasped the shunt with a needle holder and rotated it counterclockwise in order to align the spur with the incision, thereby easily removing the shunt thorough the incision site.

Ex-PRESS shunt scleral-flap implantation has, in most cases, provided resistance adequate for prevent shunt extrusion. The potential for spontaneous post-operative displacement, accordingly, is low. This means that Ex-PRESS shunt extrusion occurs more readily as a consequence of intraoperative improper fixation. In our case, a patient with senile cataract and open-angle glaucoma underwent combined surgery (cataract + Ex-PRESS shunt implantation) in both eyes. Considering that the right-eye shunt sustained good function without displacement, and keeping in mind all of the above-noted findings, we supposed that the left-eye shunt extrusion had occurred by intraoperative improper spur fixation. The determined causes of the improper spur fixation are as follows: first, the patient had low scleral rigidity originating from high myopia; second, in the left-eye operation, unlike the right-eye case, the viscoelastic material utilized in the anterior chamber was insufficient. These factors combined to compromise scleral resistance to the implanted Ex-PRESS shunt.

According to the standard procedure, when malposition or exposure on conjunctiva of the Ex-PRESS shunt occurs, the shunt is removed and secondary glaucoma surgery is performed. However, we treated a patient with impending extrusion of Ex-PRESS shunt by shunt-position adjustment. To our best knowledge, this is first case of the treatment of malpositioned EX-PRESS shunt by shunt-position adjustment. This treatment is considered to be a particularly effective method, because it renders shunt removal and secondary glaucoma surgery unnecessary.

Ex-PRESS shunt removal is always quite traumatic and in cases of impending erosion it impossible to try to reposition it instead of straight forward removal. So to prevent this situation, Ex-PRESS shunt should be inserted perpendicularly to the limbus, parallel to the iris plane and well pushed in anterior chamber for firm fixation of the spur.

## Conclusions

In conclusion, when Ex-PRESS shunt implantation is performed on a patient who shows a tendency to low scleral rigidity, care should be taken to ensure that the shunt spur is firmly fixed.

## Abbreviations

AXL: axial length
BCVA: best-corrected visual acuity
D: diopters
IOP: intraocular pressure
MMC: mitomycin C
